# Do pleural fluid pH and lactate improve classification beyond Light’s criteria?: A single-center retrospective study

**DOI:** 10.1097/MD.0000000000048606

**Published:** 2026-05-08

**Authors:** Berrin Zinnet Eraslan, Elif Yilmaz, Sümeyye Kodalak Cengiz, Sevda Cömert

**Affiliations:** a Department of Pulmonology, Kartal Dr. Lütfi Kirdar City Hospital, Istanbul, Türkiye.

**Keywords:** exudate, lactate, light’s criteria, pH, pleural effusion, ROC analysis, transudate

## Abstract

Accurate differentiation between transudative and exudative pleural effusions is essential for determining the underlying etiology and guiding management. Although Light’s criteria remain the standard diagnostic approach, their performance may be limited in borderline cases. Biochemical markers such as pleural fluid pH and lactate have been proposed as adjunct tools, yet the extent to which they provide additional diagnostic improvement beyond Light’s criteria remains unclear. This single-center retrospective study specifically evaluated whether adding pleural fluid pH and lactate to Light’s criteria improves the ability to distinguish transudative from exudative pleural effusions, quantifying the additional diagnostic value of these markers. We retrospectively analyzed 281 patients who underwent diagnostic thoracentesis between November 2020 and March 2024. Receiver operating characteristic-derived cutoff values and corresponding area under the curve (AUC)s for pH and lactate were generated and assessed within the same cohort. Multivariable logistic regression models incorporating Light’s criteria alone and in combination with pH and/or lactate were constructed to assess incremental discrimination (ΔAUC), net reclassification improvement (NRI), and integrated discrimination improvement (IDI). Of the 281 patients, 62 (22.1%) had transudative and 219 (77.9%) had exudative effusions. Pleural fluid pH ≤ 7.44 showed an AUC of 0.767 (95% CI, 0.696–0.839), sensitivity 71.4%, and specificity 70.2%. Lactate ≥ 2.05 mmol/L yielded an AUC of 0.701 (95% CI, 0.627–0.775), sensitivity 61.7%, and specificity 63.2%. Adding pH to Light’s criteria increased diagnostic accuracy (Model-2 AUC 0.985; ΔAUC + 0.010; NRI 0.904; IDI 0.035). Lactate also improved discrimination, though to a lesser extent (Model-3 AUC 0.982; ΔAUC + 0.007; NRI 0.607; IDI 0.025). Incorporating both biomarkers provided the highest incremental metrics (AUC 0.986; ΔAUC + 0.011; IDI 0.053), demonstrating a modest additive benefit across models. Pleural fluid pH and lactate, when added to Light’s criteria, provide distinct yet modest incremental gains in diagnostic performance. These findings clarify their potential as an adjunct, though **f**urther external validation is necessary before routine clinical application.

## 1. Introduction

Pleural effusion is a frequently encountered condition in pulmonology practice.^[1,2]^ It may arise from systemic diseases such as heart failure or localized pleural processes such as infection or malignancy. The initial diagnostic step involves distinguishing between transudative and exudative effusions, as this distinction directs further clinical evaluation and management. Light’s criteria, based on pleural fluid and serum protein and lactate dehydrogenase (LDH) measurements, have remained the standard diagnostic approach since their introduction in 1972.^[3]^ These criteria demonstrate a high sensitivity of approximately 98% but a lower specificity of 70% to 80%.^[4]^

Despite their widespread application, Light’s criteria have recognized limitations. In particular, patients receiving diuretic therapy may demonstrate elevated pleural fluid protein or LDH levels, potentially resulting in the misclassification of transudates as exudates.^[3]^

To overcome the limitations of Light’s criteria, the serum–pleural albumin gradient (SPAG) has been proposed as an adjunctive tool, particularly valuable in distinguishing true transudates from pseudo-exudates in heart failure, hepatic hydrothorax, and effusions with borderline biochemical values. A SPAG threshold > 1.2 g/dL has been shown to improve diagnostic classification when Light’s criteria are equivocal.^[5,6]^

Pleural fluid pH and lactate are 2 readily available biochemical parameters that can be measured using a blood gas analyzer. Pleural fluid pH has long been recognized as a clinically valuable marker, particularly in the context of complicated parapneumonic effusions and empyema, where values below 7.20 typically indicate increased metabolic activity related to bacterial infection or inflammation.^[7]^ The British Thoracic Society also recommends measuring pleural fluid pH in suspected parapneumonic effusions to guide decisions regarding chest drainage or surgical intervention.^[8]^ Similarly, previous studies have shown that pleural fluid lactate is elevated particularly in infectious and highly inflammatory exudates and may serve as a useful biomarker for the rapid diagnosis of complicated parapneumonic effusions.^[9]^ Despite several reports on lactate or pH in specific clinical settings, few have systematically evaluated their diagnostic utility in routine classification of effusions.

Given these considerations, the present study sought to address a specific clinical question:

Does the addition of pleural fluid pH and lactate to Light’s criteria improve diagnostic performance compared with Light’s criteria alone, and how does this combined approach compare with the SPAG as an established adjunct in borderline cases?

To answer this question, we evaluated the incremental diagnostic value of pH and lactate using multivariable models, receiver operating characteristic (ROC) analysis, and reclassification metrics.

## 2. Material and methods

### 2.1. Study design and setting

This retrospective observational study was conducted in the Pulmonology Department of a tertiary care center between November 2020 and March 2024. The study included adult patients undergoing diagnostic thoracentesis for pleural effusion. Only the first diagnostic thoracentesis per patient was included to avoid within-patient clustering.

### 2.2. Patient selection

Patients aged ≥18 years who underwent diagnostic thoracentesis and had available pleural fluid and serum biochemical measurements were included in the study. Exclusion criteria comprised empyema requiring urgent drainage and cases with missing essential biochemical data required for classification according to Light’s criteria. Comorbidities were not used as exclusion criteria, as the aim of the study was to evaluate pleural fluid biochemical markers in a real-world clinical population. The severity or volume of pleural effusion was not used as an inclusion criterion because the objective of the study was biochemical classification rather than radiological severity assessment. Borderline or inconclusive Light’s classifications were adjudicated using the SPAG.

### 2.3. Data collection and biochemical parameters

Demographic variables (age and sex) and major comorbidities (heart failure, malignancy, diabetes mellitus, hypertension, chronic kidney disease, and chronic lung disease) were extracted from electronic medical records. Pleural fluid measurements included LDH, total protein, albumin, glucose, pH, and lactate. Corresponding serum values for LDH, protein, albumin, and glucose were obtained from blood samples collected within 24 hours of thoracentesis.

Only laboratory and biochemical parameters required for Light’s criteria, SPAG calculation, and incremental model analyses were included. Missing data were handled by complete-case analysis without imputation. Statistical analyses were performed on de-identified datasets, and the analysts were not involved in the clinical classification process.

Pleural fluid samples for pH and lactate analysis were collected in heparinized plastic syringes, with attempts to avoid air exposure or visible hemolysis. All samples were transported immediately to the core laboratory and analyzed on a blood gas analyzer (ABL 800 FLEX, Radiometer Medical, Copenhagen, Denmark) at room temperature.

Because this was a retrospective study, precise documentation of pre-analytical factors (such as exact time from sampling to analysis, minor air exposure, and transport interval) was not available. These variations may have introduced some measurement variability in pleural fluid pH and lactate.

### 2.4. Definitions and grouping

Borderline Light’s criteria cases were defined as effusions fulfilling Light’s criteria for exudation but presenting biochemical values close to the decision thresholds or clinical features suggestive of a transudative process, such as known or suspected diuretic use or underlying heart failure. These cases were adjudicated using the SPAG, applied as an established adjunct criterion.

A SPAG value >1.2 g/dL was considered indicative of a transudative effusion. Using this predefined rule, 6 borderline cases were reclassified as transudative effusions. Sensitivity analyses were performed both including and excluding borderline Light’s criteria cases adjudicated by SPAG.

### 2.5. Statistical analysis

Data were analyzed using SPSS version 25.0 (IBM Corp., Armonk). Exudates were defined as the positive outcome in all models. ROC curves were constructed for pleural fluid pH and lactate, and optimal cutoff values were determined using the Youden index. Area under the curve (AUC) and 95% confidence intervals were calculated.

To evaluate incremental diagnostic value beyond Light’s criteria, we fitted a series of logistic regression models:

Model-1 (Light’s criteria alone),

Model-2 (Light’s + pH),

Model-3 (Light’s + lactate),

Model-4 (Light’s + pH + lactate),

Model-5 (SPAG as a continuous predictor).

The SPAG was additionally evaluated using both the empirically derived threshold of 0.804 g/dL and the conventional 1.2 g/dL cut-point. Change in AUC (ΔAUC) was computed relative to Model-1, and 95% bootstrap-derived confidence intervals were obtained. Incremental discrimination was quantified using net reclassification improvement (NRI) and integrated discrimination improvement (IDI).

Model calibration was assessed by the calibration slope, calibration intercept, and the Hosmer–Lemeshow goodness-of-fit test. Internal validation was performed using 2 nonoverlapping datasets (Dataset A and Dataset B). Missing data were handled by complete-case analysis.

Sensitivity analyses were conducted by repeating all models after excluding the 6 cases with borderline Light’s criteria that required adjudication by SPAG, and then repeating the analyses with these cases included. Additional subgroup analyses were performed within the cohort in which Light’s criteria are least reliable (borderline and adjudicated cases) to determine which adjunctive marker offers greater improvement in this clinically relevant subset. Statistical significance was defined as *P* <.05.

### 2.6. Ethical approval

The study was approved by the Ethics Committee of Kartal Dr Lütfi Kirdar City Hospital (Decision No: 2024/010.99/5/1; Date: June 28, 2024) and conducted in accordance with the principles of the Declaration of Helsinki. Informed consent was waived due to the retrospective design.

## 3. Results

The baseline demographic and clinical characteristics of the study population are summarized in Table 1.

**Table 1:** Baseline characteristics of the study population.

Variables	Total (n = 281)
Female n %	109 (38.8)
Male n %	172 (61.2)
Age, years, median (IQR)	67 (54.5–75)
Patients with ≥1 comorbidity, n (%)	242 (86.1)
Hypertension n %	139 (49.5)
Diabetes mellitus, n %	83 (29.5)
Congestive heart failure, n%	46 (16.4)
Coronary artery disease, n %	64 (22.8)
Chronic obstructive pulmonary disease n %	53 (18.9)
Chronic kidney disease, n %	45 (16.0)
Malignancy, n %	130 (46.3)

IQR = interquartile range.

**Figure 1.**
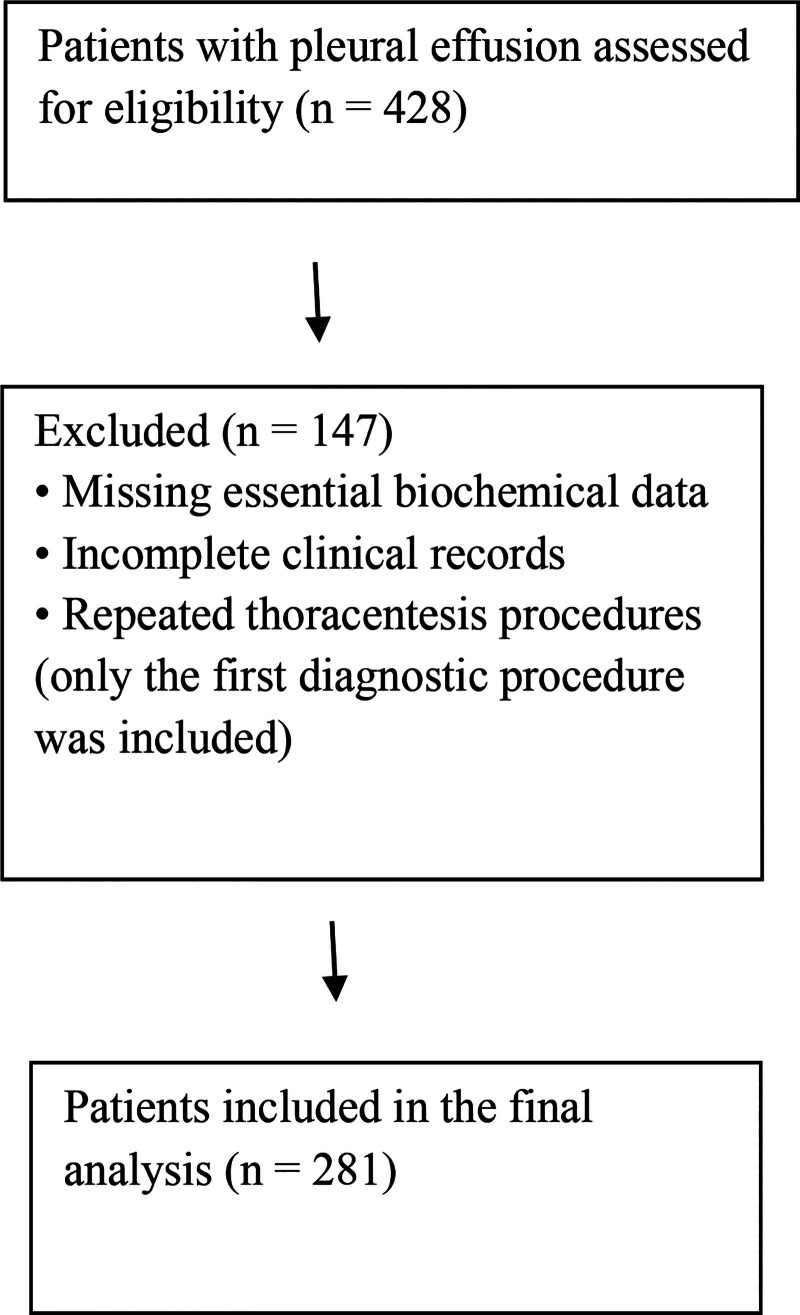
Flow diagram of patient selection and study inclusion.

A total of 428 patients with pleural effusion were assessed for eligibility. After excluding patients with missing essential biochemical data, incomplete clinical records, and repeated thoracentesis procedures, 281 patients were included in the final analysis (Fig. 1). Of these, 62 (22.1%) were classified as transudates and 219 (77.9%) as exudates according to Light’s criteria. Six borderline Light’s criteria cases were adjudicated using the SPAG and were reclassified as transudates.

### 3.1. Diagnostic performance of pleural fluid pH and lactate

**Figure 2.**
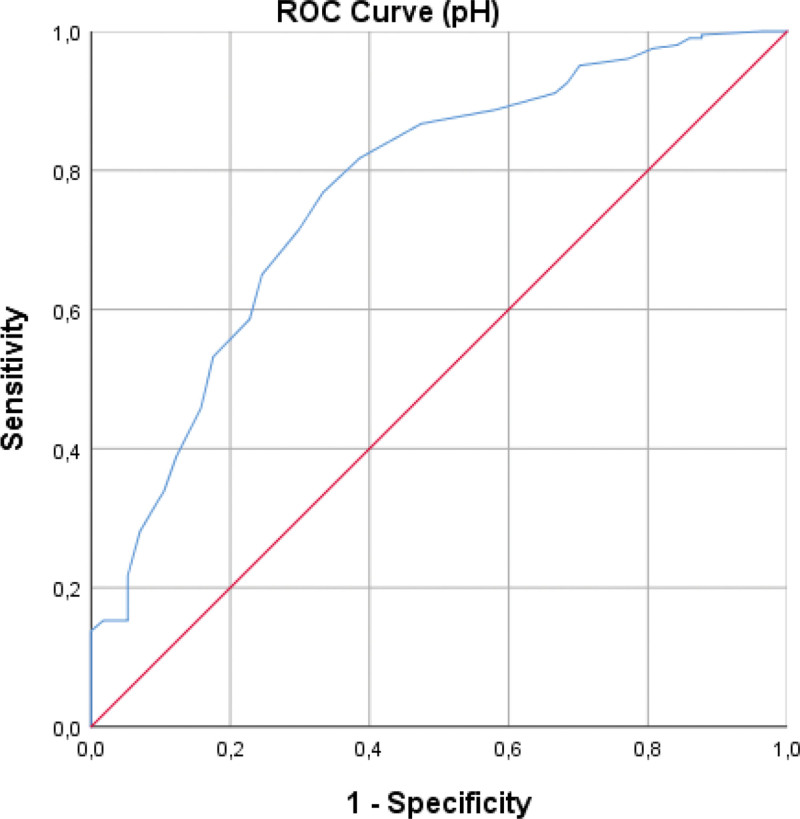
ROC curve for pleural fluid pH. pH = hydrogen ion concentration, ROC = receiver operating characteristic.

**Figure 3.**
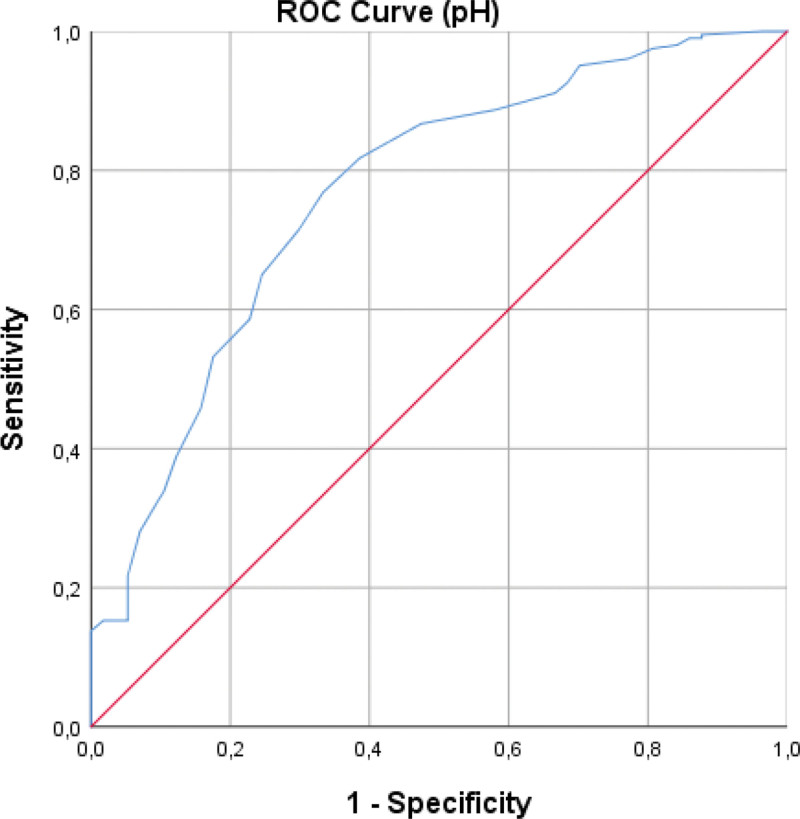
ROC curve for pleural fluid lactate. ROC = receiver operating characteristic.

ROC analyses demonstrated that pleural fluid pH and lactate significantly differentiated transudates from exudates (Table 2). The optimal pH cutoff was ≤7.44, yielding an AUC of 0.767 (95% CI: 0.696–0.839), sensitivity 71.4%, and specificity 70.2% (Fig. 2). The optimal lactate cutoff was ≥2.05 mmol/L, with an AUC of 0.701 (95% CI: 0.627–0.775), sensitivity 61.7%, and specificity 63.2% (Fig. 3). Overall, pH demonstrated superior discrimination compared with lactate.

**Table 2:** Diagnostic performance of pleural fluid ph and lactate based on ROC analysis.

	pH	Lactate
Cutoff value	≤7.44	≥2.05
AUC (95% CI)	0.767 (0.696–0.839)	0.701 (0.627–0.775)
Sensitivity (95% CI)	71.43% (64.68–77.53)	61.69% (54.59–68.44)
Specificity (95% CI)	70.18 %(56.60–81.57)	63.16% (49.34–75.55)
Youden index	41.61	24.85

AUC = area under the curve, CI = confidence interval, ROC = receiver operating characteristic.

### 3.2. Incremental value beyond Light’s criteria: primary analysis (borderline cases excluded)

Predictive models for distinguishing transudates from exudates were constructed in the full cohort excluding borderline SPAG-adjudicated cases (n = 273). The baseline model using Light’s criteria (Model-1) showed excellent discrimination (AUC 0.975, 95% CI: 0.959–0.991).

Adding pH to Light’s criteria (Model-2) improved diagnostic performance (AUC 0.985, ΔAUC + 0.010). This model yielded a substantial net reclassification improvement (NRI 0.904) and a significant integrated discrimination improvement (IDI 0.035; *P* = .033). These findings indicate that pH provided a statistically significant but modest improvement in classification accuracy, particularly among misclassified cases.

Adding lactate alone (Model-3) resulted in a smaller but statistically significant improvement (AUC 0.982, ΔAUC + 0.007; NRI 0.607; IDI 0.025; *P* = .039).

The combined model, including both pH and lactate (Model-4), provided the highest overall performance (AUC 0.986, ΔAUC + 0.011). Although both biomarkers contributed, model improvements were predominantly driven by pH, as reflected by a higher NRI (0.880) and IDI (0.053) values, with pH remaining statistically significant (*P* = .019).

The SPAG model (Model-5), analyzed as a continuous variable, demonstrated an AUC of 0.896, and an empirically optimized cutoff of 0.804 g/dL yielded a sensitivity of 83.7%. However, its overall performance remained lower than the Light’s + pH model, consistent with its role as an adjunct comparator rather than a replacement strategy (Table 3).

**Table 3:** Logistic regression models excluding borderline cases.

	OR	95% CI	*P*
Model-1			
Pleural fluid/serum protein ratio > 0.5	49.230	12.033–201.412	<.001
Pleural fluid/serum LDH ratio > 0.6	48.446	11.480–204.447	<.001
Pleural fluid LDH level > two-thirds of the upper limit of normal serum LDH	11.980	1.516–94.656	.019
Constant	6.778	–	<.001
Model AUC (95% CI) = 0.975 (0.959–0.991)			
Model-2			
Pleural fluid/serum protein ratio > 0.5	41.673	8.990–193.184	<.001
Pleural fluid/serum LDH ratio > 0.6	59.388	11.341–311.005	<.001
Pleural fluid LDH level > two-thirds of the upper limit of normal serum LDH	13.443	1.363–132.616	.026
pH	0.000	0.000–0.033	.008
Constant	9.763	0.07	–
Model AUC (95% CI) = 0.985 (0.973–0.996) ΔAUC = 0.010 NRI = 0.904 IDI = 0.035			
Model-3			
Pleural fluid/serum protein ratio > 0.5	64.733	12.724–339.583	<.001
Pleural fluid/serum LDH ratio > 0.6	40.389	9.09–180.466	<.001
Pleural fluid LDH level > two-thirds of the upper limit of normal serum LDH	8.152	0.862–77.098	.067
Lactate	2.36	1.046–5.329	.039
Constant	0.803	–	.834
Model AUC (95% CI) = 0.982 (0.969–0.995) ΔAUC = 0.007 NRI = 0.607 IDI = 0.025			
Model-4			
Pleural fluid/serum protein ratio > 0.5	70.634	11.714–425.907	<.001
Pleural fluid/serum LDH ratio > 0.6	57.620	10.120–328.051	<.001
Pleural fluid LDH level > two-thirds of the upper limit of normal serum LDH	11.462	1.066–123.233	.044
pH	0.000	0.000–0.126	.019
Lactate	2.423	0.955–6.150	.063
Constant	6.548	–	.019
Model AUC (95% CI) = 0.986 (0.975–0.997) ΔAUC = 0.011 NRI = 0.880 IDI = 0.053			
Model-5			
SPAG	0.026	0.009–0.073	<.001
Constant	826.533	<0.001	–
Model AUC (95% CI) = 0.896 (0.848–0.943); SPAG cut off = 0.804 (sensitivity = 83.7%)			

AUC = area under the curve, CI = confidence interval, IDI = integrated discrimination improvement, LDH = lactate dehydrogenase, NRI = net reclassification improvement, pH = hydrogen ion concentration, ROC = receiver operating characteristic, SPAG = serum–pleural albumin gradient.

### 3.3. Sensitivity analysis (borderline Light’s criteria cases included)

When borderline Light’s cases adjudicated by SPAG were included (n = 281), the overall pattern remained consistent. The AUCs for Model-2, Model-3, and Model-4 were 0.981, 0.981, and 0.983, respectively, with ΔAUC values ranging from + 0.008 to + 0.010. NRI and IDI again demonstrated that pH contributed more substantially than lactate to incremental discrimination (Table 4).

**Table 4:** Logistic regression models including borderline cases.

	OR	95% CI	*P*
Model-2			
Pleural fluid/serum protein ratio > 0.5	29.242	6.989–122.874	<0.001
Pleural fluid/serum LDH ratio > 0.6	63.405	13.638–294.786	<0.001
Pleural fluid LDH level > two-thirds of the upper limit of normal serum LDH	12.767	1.405–115.977	0.024
pH	0.000	0.000–0.310	0.026
Constant	3.779	–	0.023
Model AUC (95 %CI) = 0.981 (0.968–0.994) ΔAUC = 0.008 NRI = 0.553 IDI = 0.022			
Model-3			
Pleural fluid/serum protein ratio >0.5	55.264	10.921–279.666	<0.001
Pleural fluid/serum LDH ratio >0.6	47.539	10.937–206.637	<0.001
Pleural fluid LDH level > two-thirds of the upper limit of normal serum LDH	8.635	0.901–82.777	0.062
Lactate	2.405	1.092–5.300	0.029
Constant	0.708	–	0.736
Model AUC (95% CI) = 0.981 (0.967–0.994) ΔAUC = 0.008 NRI = 0.616 IDI = 0.027			
Model-4			
Pleural fluid/serum protein ratio >0.5	50.919	9.485–273.345	<0.001
Pleural fluid/serum LDH ratio >0.6	60.324	12.111–300.473	<0.001
Pleural fluid LDH level > two-thirds of the upper limit of normal serum LDH	10.682	1.044–109.309	0.046
pH	0.000	0.000–1.514	0.061
Lactate	2.538	1.059–6.083	0.037
Constant	1.387	–	0.063
Model AUC (95% CI) = 0.983 (0.970–0.995) ΔAUC = 0.010 NRI = 0.877 IDI = 0.044			
Model-5			
SPAG	0.030	0.012–0.080	<0.001
Constant	582.901	<0.001	
Model AUC (95% CI) = 0.885 (0.836–0.934); SPAG cut off = 0.804 (Sensitivity = 83.7%)			

AUC = area under the curve, CI = confidence interval, IDI = integrated discrimination improvement, LDH = lactate dehydrogenase, NRI = net reclassification improvement, pH = hydrogen ion concentration, ROC = receiver operating characteristic, SPAG = serum–pleural albumin gradient.

These findings indicate that the added value of pH is robust regardless of whether borderline cases are adjudicated or fully included.

### 3.4. Model calibration and internal validation

Calibration analyses showed that all models achieved good agreement between predicted and observed probabilities, with all Hosmer–Lemeshow *P*-values >.6 (Table 5). Calibration slopes were close to 1 in both datasets, indicating excellent calibration and minimal overfitting. Model-4 (Light’s + pH + lactate) demonstrated the best calibration performance.

**Table 5:** Results of calibration analyses for diagnostic models.

Dataset B	Light’s criteria alone	Light’s criteria with biomarker(s)
Model (1–2)		
Calibration slope	0.989	0.999
Calibration intercept	0.043	0.000
Hosmer–Lemeshow *P*-value	.999	.998
Model (1–3)		
Calibration slope	0.981	1.000
Calibration intercept	0.040	0.000
Hosmer–Lemeshow *P*-value	.999	.989
Model (1–4)		
Calibration slope	0.981	1.000
Calibration intercept	0.031	0.000
Hosmer–Lemeshow *P*-value	.999	.851
Dataset A		
Model (1–2)		
Calibration slope	0.977	0.975
Calibration intercept	0.010	0.007
Hosmer–Lemeshow *P*-value	.843	.998
Model (1–3)		
Calibration slope	0.977	0.975
Calibration intercept	0.010	0.010
Hosmer–Lemeshow *P*-value	.982	.994
Model (1–4)		
Calibration slope	0.977	0.973
Calibration intercept	0.010	0.006
Hosmer–Lemeshow *P*-value	.999	.954

Models represent Light’s criteria alone and Light’s criteria combined with pleural fluid pH and/or lactate.

pH = hydrogen ion concentration.

**Figure 4.**
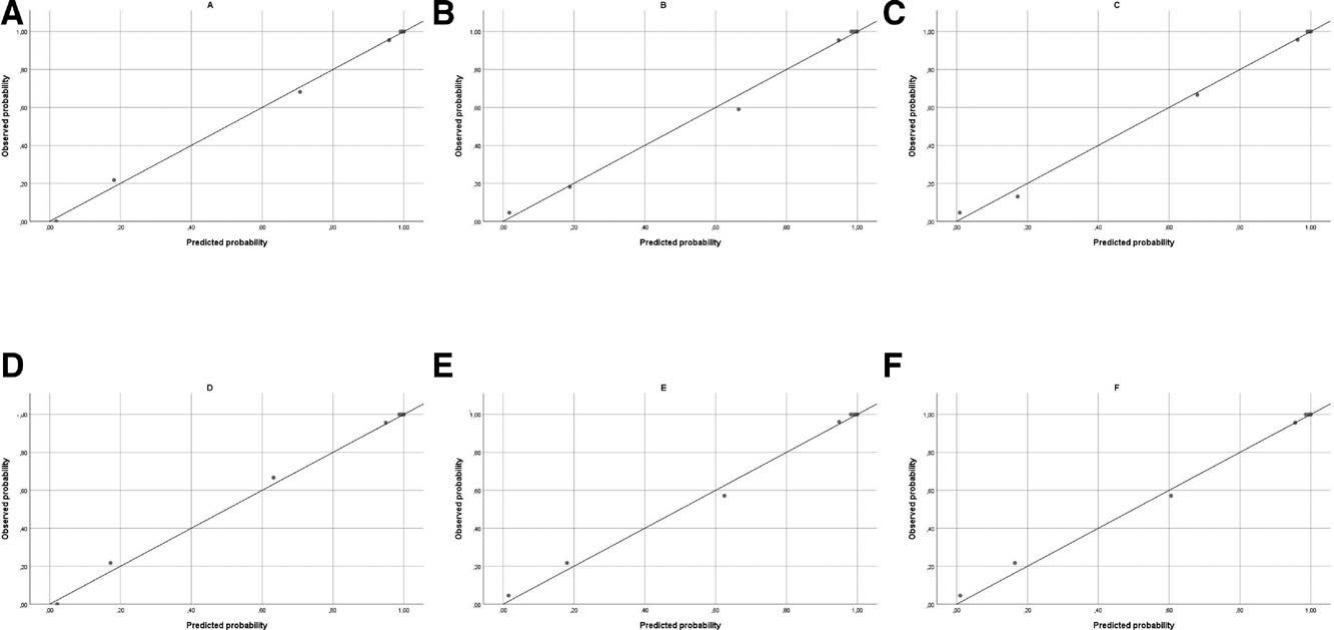
Calibration plots for logistic regression models.

Internal validation using 2 nonoverlapping datasets (Dataset A and Dataset B) confirmed the stability of the calibration slopes, intercepts, and Hosmer–Lemeshow *P*-values, supporting the robustness of the models (Fig. 4). The validation process reduced the risk of overfitting and confirmed that the effect of adding pH and lactate was consistent across data subsets.

### 3.5. Graphical summary of model performance

**Figure 5.**
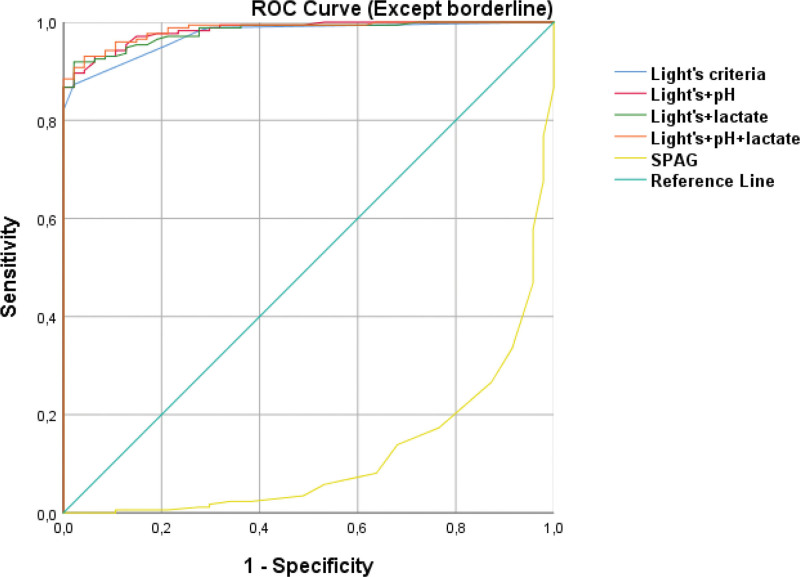
ROC curves for all models in patients excluding borderline cases. ROC = receiver operating characteristic.

**Figure 6.**
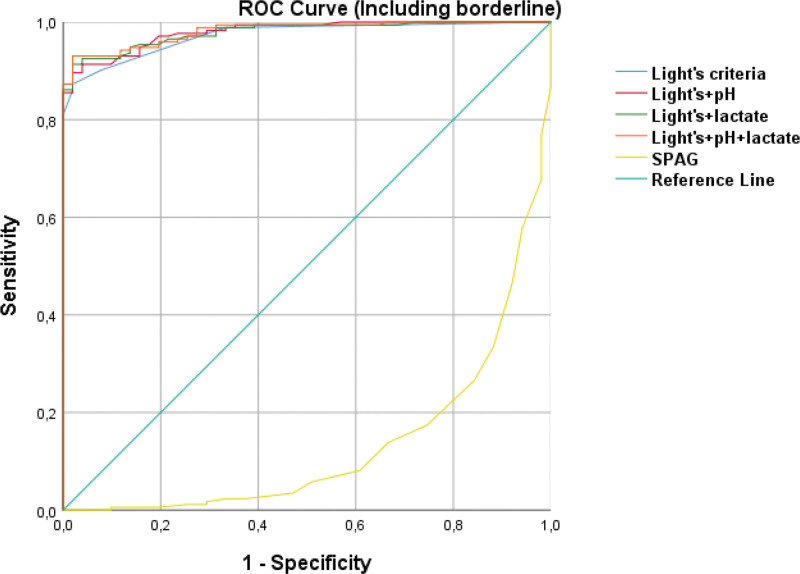
ROC curves for all models in patients including borderline cases. ROC = receiver operating characteristic.

ROC curves comparing logistic regression models based on Light’s criteria alone, Light’s criteria combined with pleural fluid pH and/or lactate, and the SPAG model are presented in Figures 5 and 6.

## 4. Discussion

This retrospective study examined the diagnostic performance of pleural fluid pH and lactate and assessed whether these biomarkers provide incremental value beyond Light’s criteria in differentiating transudative from exudative pleural effusions. Although both markers showed statistically significant differences between groups, their overall discriminative ability was modest, and although statistically significant incremental improvements were observed, these did not translate into substantial clinical impact beyond Light’s criteria was modest and did not meaningfully improve classification when added to Light’s criteria.

Pleural fluid pH demonstrated only moderate discriminatory ability for distinguishing transudates from exudates (AUC 0.767), which is consistent with previously reported performance ranges. Early foundational work by Good et al showed that markedly low pleural fluid pH values (<7.30) occur exclusively in exudative effusions – particularly empyema, malignancy, and tuberculosis – highlighting pH as a marker of pleural inflammation rather than a general classifier of effusion type.^[10]^ Consistent with this evidence, the 2023 BTS Pleural Guidelines recommend measuring pleural pH primarily to identify complicated parapneumonic effusions and guide drainage decisions, rather than to differentiate transudates from exudates in routine practice.^[8]^ In contrast to these earlier cohorts enriched with highly inflammatory exudates, our study included very few such cases and no empyema, which likely explains the absence of markedly low pH values and the moderate discriminative performance observed (AUC 0.767). This narrower physiological range, combined with unavoidably variable pre-analytical conditions inherent to retrospective data – as also emphasized by Rahman et al – reduces the reliability of pH measurement and may account for its limited incremental value beyond Light’s criteria in our cohort.^[11]^

In our study, pleural fluid lactate demonstrated a modest diagnostic performance in distinguishing exudates from transudates and did not provide a meaningful incremental contribution beyond Light’s criteria. This finding is partly consistent with the study by Porta et al, who reported high discriminative ability for exudates (AUC 0.85); variations in the clinical spectrum and patient distribution may explain differences in lactate performance across studies.^[12]^ Similarly, Kho et al showed that lactate can exhibit strong diagnostic power in specific exudative conditions, such as complicated parapneumonic effusions.^[13]^ In another study, the authors showed that pleural fluid lactate levels were highest in infectious effusions and could reliably distinguish infectious from noninfectious pleural disease.^[14]^ In our cohort, the heterogeneous nature of the exudate group and the lack of subclassification of underlying etiologies may have limited the ROC performance of lactate and prevented it from offering additional diagnostic value alongside Light’s criteria.

When overall model performance is considered, adding pleural fluid pH and lactate appears to provide only limited incremental value beyond Light’s criteria. Although both biomarkers demonstrated moderate individual discriminatory ability, their inclusion resulted in minimal improvements in model accuracy, as reflected by the small ΔAUC values and the modest gains in NRI and IDI. These findings suggest that the high baseline performance of Light’s criteria leaves limited room for substantial enhancement by additional biochemical parameters. Furthermore, the heterogeneity of the exudate group and the absence of etiologic subclassification may have contributed to variability in biomarker behavior, reducing their additive diagnostic impact. Although pH and lactate yielded statistically significant incremental improvements in AUC, NRI, and IDI, the clinical impact of these gains remains limited due to the already excellent baseline performance of Light’s criteria. Taken together, these results indicate that pH and lactate function as biologically plausible adjuncts, providing only limited adjunctive augmentation beyond Light’s criteria in routine exudate–transudate classification.

This study has several limitations. First, its retrospective, single-center design may limit the generalizability of the findings. Second, etiologic subclassification of exudative effusions was unavailable, preventing assessment of whether pH and lactate differ across specific exudative subgroups, such as malignant, tuberculous, or parapneumonic effusions. Third, pH and lactate measurements were obtained at a single time point using routine laboratory methods, which may be subject to pre-analyticalvariability and lack of standardization. In addition, the high baseline discriminatory performance of Light’s criteria may have limited the ability to demonstrate meaningful incremental improvements in AUC, NRI, or IDI when additional biomarkers were incorporated. Finally, external validation was not performed, and future prospective multicenter studies are needed to confirm the reproducibility and clinical utility of these findings.

## 5. Conclusion

In this retrospective study, pleural fluid pH and lactate demonstrated statistically significant incremental improvements in discrimination metrics such as NRI and IDI when added to Light’s criteria, indicating measurable enhancement in model-level classification. However, the clinical impact of these gains remains modest, largely due to the already excellent baseline performance of Light’s criteria. Overall, pH and lactate appear to function as biologically plausible adjuncts, providing limited but quantifiable adjunctive diagnostic value without altering routine exudate–transudate classification.

## Acknowledgments

We gratefully acknowledge the valuable support provided by Dr Güllü Eren (Public Health Specialist) during the statistical analysis phase of this study. Her expertise and assistance contributed significantly to the rigor of the statistical evaluation.

## Author contributions

**Conceptualization:** Berrin Zinnet Eraslan, Sümeyye Kodalak Cengiz.

**Data curation:** Berrin Zinnet Eraslan, Elif Yilmaz, Sümeyye Kodalak Cengiz.

**Formal analysis:** Elif Yilmaz, Sevda Cömert.

**Methodology:** Berrin Zinnet Eraslan, Elif Yilmaz, Sevda Cömert.

**Writing – original draft:** Berrin Zinnet Eraslan, Sevda Cömert.

**Writing – review & editing:** Berrin Zinnet Eraslan, Sümeyye Kodalak Cengiz, Sevda Cömert.
